# Survival Prediction of Patients Treated With Immune Checkpoint Inhibitors *via* KRAS/TP53/EGFR-Single Gene Mutation

**DOI:** 10.3389/fphar.2022.878540

**Published:** 2022-03-23

**Authors:** Shui Liu, Shuai Geng, Ning Shi, Lili Zhang, Wenxin Xue, Yiwen Li, Kai Jiang

**Affiliations:** ^1^ Department of Pharmacy, Emergency General Hospital, Beijing, China; ^2^ Department of Pharmacy, Strategic Support Force Medical Center, Beijing, China; ^3^ Department of Gastroenterology, Traditional Chinese Medical Hospital of Zhuji, Zhuji, China

**Keywords:** immune checkpoint inhibitors, cancer, gene mutation, progression free survival, overall survival, survival benefit

## Abstract

**Background:** Immune checkpoint inhibitors (ICIs) have become an effective treatment option for cancer. KRAS, EGFR and TP53 are common mutated oncogenes in cancer whose single gene status may predict the therapeutic effect of clinical ICIs. In this efficacy evaluation, we aimed to clarify whether the single gene mutation status of KRAS, EGFR or TP53 affects the survival benefits of ICIs in cancer patients.

**Methods:** We used PubMed, Cochrane Library, web of science, and clinical trials Gov database to retrieve qualified documents, the time was up to January 2022. Hazard ratios (HRS) and 95% confidence intervals (CIs) were used to determine the single gene mutation status and no progression of KRAS, EGFR or TP53.

**Results:** A total of 19 studies included 7029 cancer patients treated with ICIs. The results showed that KRAS, EGFR or TP53 single gene mutation could significantly improve PFS and OS in patients receiving ICIs, but the degree of improvement was different. The risk of prolongation of PFS (HR = 1.48, 95% CI = 1.19-1.85, *p* = 0.0004) and OS (HR = 1.68, 95% CI = 1.36-2.07, *p* < 0.00001) caused by TP53 single gene mutation was relatively high, the risk ratio of prolongation of PFS (HR = 1.38, 95% CI = 1.21-1.57, *p* < 0.00001) and OS (HR = 1.56, 95% CI = 1.20-2.04, *p* = 0.001) caused by EGFR single gene mutation was the second, the risk ratio of prolongation of PFS (HR = 1.33, 95% CI = 1.12-1.57, *p* = 0.001) and OS (HR = 1.39, 95% CI = 1.18-1.63, *p* < 0.00001) caused by KRAS single gene mutation was relatively low, and the results were significantly different.

**Conclusion:** In cancer patients, KRAS, EGFR or TP53 single gene status is correlated with the benefits of immunotherapy PFS and OS, which suggests that gene sequencing should be carried out in time in the process of clinical treatment to determine the gene mutation of patients and better predict the clinical treatment effect of ICIs.

## Introduction

With the advent of the era of tumor immunity, significant progress has been made in the treatment of cancers. In recent years, immune checkpoint inhibitors have become an effective treatment option and means for the treatment of cancer (Ferlay et al., 2015). Programmed death ligand 1 (PD-L1) is an immune checkpoint protein expressed on cancer cells or cancer infiltrating immune cells. PD-L1 binds to the programmed death 1 (PD-1) receptor on activated T cells and induces tumor immune escape by down regulating the function of antitumor T cells (Alexandrov et al., 2013; Brahmer et al., 2015). Therefore, inhibition of PD-1/PD-L1 pathway can induce immune response to cancer by restoring T cell activity (Moore et al., 2020).

Immune checkpoint inhibitors (ICIs) refer to antibodies against PD-1/PD-L1, which block the inhibition signal mediated by PD-1/PD-L1 (Dong et al., 2017). Many clinical trials involving patients with cancer tumors have shown that ICIs can achieve better survival outcomes than standard chemotherapy (Ozaki et al., 2020).

Tumor mutation load has been considered as a potential marker of cancer response to ICIs. High mutation load may be related to the increase of new antigens recognized by T cells to increase antitumor T cell response (Dong et al., 2017). KRAS, EGFR or TP53 are the most common mutated oncogenes in cancer (Dong et al., 2017). However, it is unclear whether the efficacy of ICIs in cancer patients is related to KRAS, EGFR or TP53 single gene mutations. We conducted this meta-analysis to investigate whether KRAS, EGFR or TP53 single gene mutation status affects the survival benefits of ICIs in cancer patients.

## Materials and Methods

Systematically search the domestic and foreign literature on the efficacy of ICIs in cancer patients and KRAS, EGFR or TP53 single gene mutations, and evaluate whether KRAS, EGFR or TP53 single gene mutations affect the survival benefits of ICIs in cancer patients.

### Search Strategy

We performed multiple retrieval tools: 1) Computer literature database search: 1) Retrieval of computer literature database: ① Chinese search terms include immune checkpoint inhibitors, cancer, gene mutation, etc; ② English search terms include ICIS, cancer, KRAS, EGFR, TP53, etc; ③ PubMed, Cochrane Library, EMBASE and EBSCO evidence-based medicine databases are searched in different combinations. The search items include title, abstract and keywords. The search time limit is from the establishment of the database to January 2022. 2) Manual retrieval of ASCO conference related literature as a supplement to computer retrieval.

### Study Selection

Immunotherapy is used more and more in many cancer patients. Some research results show that KRAS, EGFR or TP53 single gene mutation leads to the reduction of PFS and OS in cancer patients using ICIs. In order to further systematically evaluate the correlation between gene mutation and survival benefit of ICIs patients, We used inclusion and exclusion criteria for this regimen: inclusion criteria were: 1) confirmed solid tumor; 2) Overall survival (OS) or progressionfree survival (PFS) data can be used to evaluate the efficacy of immune checkpoint inhibitors; 3) KRAS EGFR or TP53 single gene mutation status; 4) Literature type: prospective or retrospective studies, randomized controlled trials, whether blind or lacking, also included animal studies reviews editorial reviews or case reports were excluded duplicative studies, unbalanced matching procedures, or incomplete data were excluded.

### Data Extraction and Quality Assessment

We used the Newcastle Ottawa scale (NOS) to evaluate the quality of the included studies (Lazarus et al., 2019). The inclusion studies were evaluated according to the following criteria: 1) whether they were representative; 2) determination of blind method; 3) whether the random method is determined; 4) Completeness of outcome events; 5) Comparability of included studies; 6) Assessment of outcome events; 7) Whether there is follow-up; 8) Completeness of follow-up. High quality literature is rated 7-9, medium quality literature is rated 4-6, and low quality literature is rated 3 or lower. Data were extracted independently by two reviewers according to specified selection criteria. Differences of opinion are resolved by discussion between authors or by obtaining input from a third evaluator.

### Statistical Analysis

We extracted key data from the preliminary study and analyzed it using Review Manager 5.4. The results are expressed as odd risk with 95% confidence interval (CI), and the continuous results are expressed as weighted mean difference. In the absence of statistical heterogeneity, fixed effect models were used to aggregate data. If there is statistical heterogeneity (*p* < 0.05, *I* ^*2*^ ≥ 50%), the random effect model is used.

## Results

### Search Results and Patient Characteristics

**FIGURE 1:**
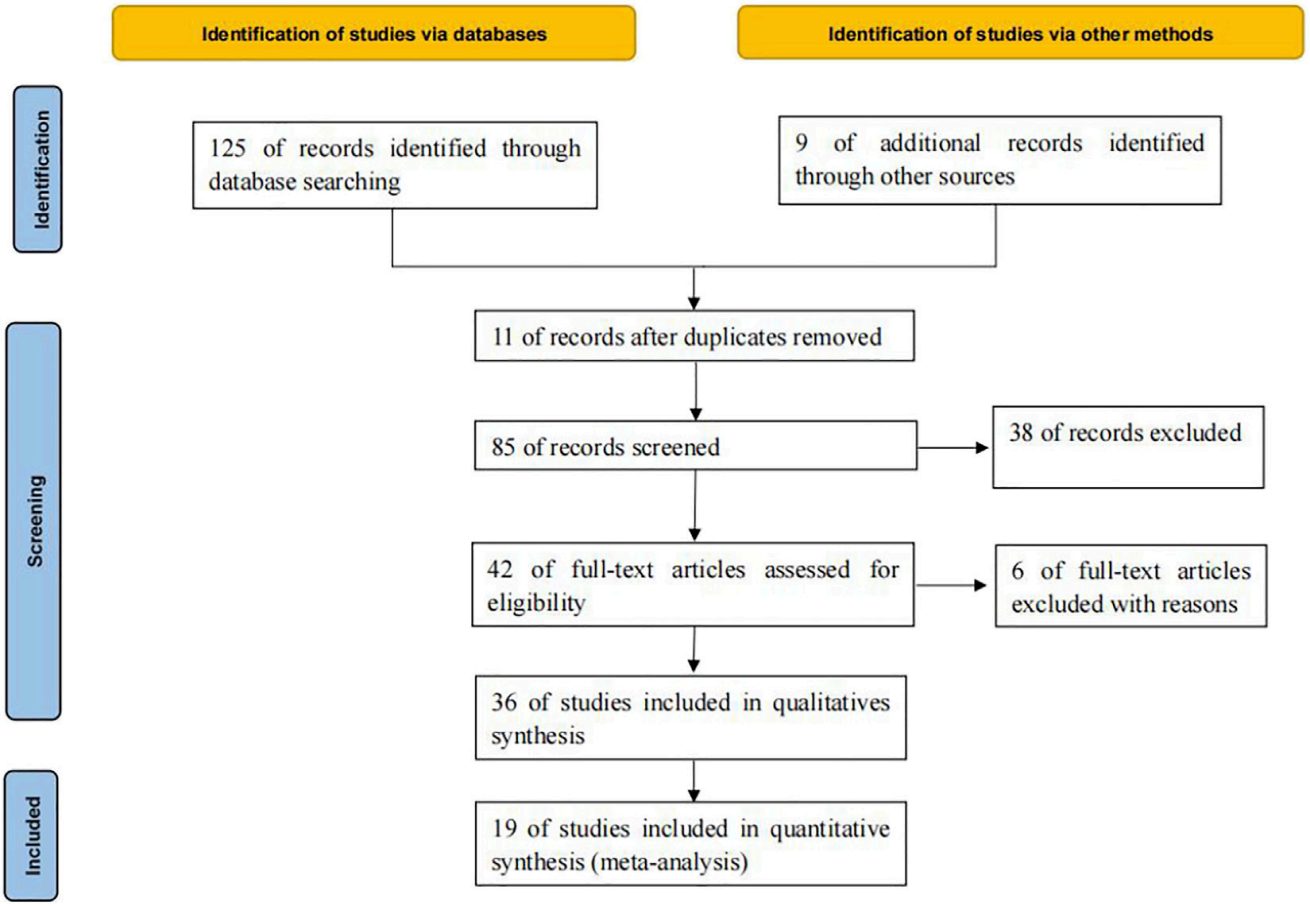
PRISMA Flow chart of article selection.

125 relevant literatures were obtained through database retrieval, excluding 90 duplicates, case reports, reviews and irrelevant contents. 36 literatures were screened in strict accordance with the above screening process. Finally, 19 (Facchinetti et al., 2017; Fujimoto et al., 2018; Garassino et al., 2018; Lin et al., 2018; Ahn et al., 2019; Assoun et al., 2019; Cho et al., 2019; Guibert et al., 2019; Jeanson et al., 2019; Ng et al., 2019; Gianoncelli et al., 2020; Lyu et al., 2020; Marinelli et al., 2020; Morita et al., 2020; Motzer et al., 2020; Schoenfeld et al., 2020; Xiang et al., 2020; An et al., 2021; Kartolo et al., 2021) studies were included, which met the quantitative analysis, involving 7029 cancer patients, as shown in Figure 1.

A total of 7029 cancer patients who met the requirements were included in 19 literatures, including 3058 patients with KRAS, EGFR or TP53 single gene mutation and 3971 patients without this gene mutation. All 19 literatures are high-quality literatures, as shown in Tables 1, 2, 3.

**TABLE 1:** Basic characteristics of included studies with KRAS status.

Author	Year	Type of Cancer	Treatment	Line	KRAS Status	NO.of Patients	HR for PFS(95%CI)	*p*-Value for PFS	HR for OS(95%CI)	*p*-Value for OS	Quality
Luo CL (Xiang et al., 2020)	2020	LUAD	PD-1/PD-L1 inhibitor	First line	MT	207	NA	NA	1.515(1.172-1.960)	0.0015	7
					WT	539					
Adi Kartolo (Kartolo et al., 2021)	2021	NSCLC	Nivolumab	First line	MT	54	1.184(0.571-2.455)	0.651	0.901(0.417-1.946)	0.791	7
					WT	24					
Arnaud Jeanso (Jeanson et al., 2019)	2019	NSCLC	PD-1/PD-L1 inhibitor	First line	MT	162	0.93 (0.71-1.21)	0.584	0.93(0.68-1.29)	0.682	7
					WT	93					
Lin SY (Lin et al., 2018)	2018	NSCLC	Nivolumab, Pembrolizumab	First line	MT	10	0.73(0.32-1.66)	0.457	1.28(0.49-3.38)	0.614	7
					WT	30					
Terry L. Ng (Ng et al., 2019)	2018	NSCLC	PD-1/PD-L1 inhibitor	First line	MT	77	0.502(0.303-0.806)	0.004	NA	NA	7
					WT	112					
Nicolas Guibert (Guibert et al., 2019)	2019	NSCLC	PD-1/PD-L1 inhibitor	Second line	MT	31	0.46	0.11	NA	NA	8
					WT	66					
Letizia Gianoncelli (Gianoncelli et al., 2020)	2020	NSCLC	PD-1/PD-L1 inhibitor	First line	MT	43	0.89 (0.59-1.34)	0.58	0.81 (0.50-1.31)	0.38	7
					WT	117					
Adam J. Schoenfeld (Schoenfeld et al., 2020)	2020	LUAD	PD-1/PD-L1 inhibitor	First line	MT	527	0.42(0.31-0.60)	<0.001	0.50(0.31-0.80)	0.003	7
					WT	1059					
D. Marinelli (Marinelli et al., 2020)	2020	LUAD	PD-1/PD-L1 inhibitor	First line	MT	26	NA	NA	1.40(0.85-2.31)	0.188	7
					WT	173					
Josiah An (An et al., 2021)	2021	NSCLC	PD-1/PD-L1 inhibitor	First line	MT	14	1.10(0.50-2.42)	0.81	NA	NA	7
					WT	56					

LUAD, lung adenocarcinoma; NSCLC, Non-small cell lung cancer; PFS, progression free survival; OS,overall survival; MT, mutant-type; WT, wild-type; HR,Hazard ratio; NA, Not available.

**TABLE 2:** Basic characteristics of included studies with TP53 status.

Author	Year	Type of Cancer	Treatment	Line	TP53 Status	No.of Patients	HR for PFS(95%CI)	*p*-Value for PFS	HR for OS(95%CI)	*p*-Value for OS	Quality
Luo CL (Xiang et al., 2020)	2020	LUAD	PD-1/PD-L1 inhibitor	First line	MT	84	NA	NA	1.618(1.128-2.505)	0.0108	7
					WT	123					
Nicolas Guibert (Guibert et al., 2019)	2019	NSCLC	PD-1/PD-L1 inhibitor	Second line	MT	31	NA	NA	0.36	0.011	7
					WT	66					
Adam J. Schoenfeld (Schoenfeld et al., 2020)	2020	LUAD	PD-1/PD-L1 inhibitor	First line	MT	701	0.62(0.44-0.89)	0.006	0.65(0.43-0.99)	0.04	7
					WT	885					
Josiah An (An et al., 2021)	2021	NSCLC	PD-1/PD-L1 inhibitor	First line	MT	21	1.02(0.52-1.99)	0.96	NA	NA	7
					WT	49					
Qiong Lyu (Lyu et al., 2020)	2020	BLCA	PD-1/PD-L1 inhibitor	First line	MT	99	NA	NA	0.65(0.44-0.99)	0.041	7
					WT	111					
Sandra Assoun (Assoun et al., 2019)	2019	NSCLC	Nivolumab	NA	MT	41	0.53(0.30-0.95)	0.03	0.48(0.25-0.95)	0.04	7
					WT	31					
Robert J. Motzer-1 (Motzer et al., 2020)	2019	RCC	Avelumab	First line	MT	27	0.96 (0.51-1.83)	0.9065	NA	NA	7
					WT	329					
Robert J. Motzer-2 (Motzer et al., 2020)	2019	RCC	Avelumab	First line	MT	39	1.47 (0.95-2.27)	0.0858	NA	NA	7
					WT	336					

LUAD, lung adenocarcinoma; NSCLC, Non-small cell lung cancer; BLCA, bladder cancer; RCC, renal cell carcinoma; PFS, progression free survival; OS, overall survival; MT, mutant-type; WT, wild-type; HR, Hazard ratio; NA, Not available.

**TABLE 3:** Basic characteristics of included studies with EGFR status.

Author	Year	Type of Cancer	Treatment	Line	EGFR Status	No.of Patients	HR for PFS(95%CI)	*p*-Value for PFS	HR for OS(95%CI)	*p*-Value for OS	Quality
Lin SY (Lin et al., 2018)	2018	NSCLC	Nivolumab, Pembrolizumab	First line	MT	25	2.00(1.11-3.62)	0.022	1.07(0.50-2.26)	0.867	7
					WT	49					
Adam J. Schoenfeld (Schoenfeld et al., 2020)	2020	LUAD	PD-1/PD-L1 inhibitor	First line	MT	465	0.75(0.38-1.48)	0.36	0.79(0.36-1.73)	0.53	7
					WT	1121					
Francesco (Facchinetti et al., 2017)	2017	NSCLC	PD-1/PD-L1 inhibitor	First line	MT	NA	NA	NA	0.45(0.26-0.81)	0.002	7
					WT	NA					
Marina Chiara Garassino (Garassino et al., 2018)	2018	NSCLC	Nivolumab	First line	MT	102	1.38 (1.11-1.72)	0.004	1.11 (0.84-1.47)	0.46	7
					WT	1293					
Ryo Morita (Morita et al., 2020)	2020	NSCLC	Nivolumab	First line	MT	116	1.11 (0.84-1.45)	0.4634	1.74(1.41-2,15)	<0.0001	7
					WT	641					
Beung-Chul Ahn (Ahn et al., 2019)	2019	NSCLC	PD-1/PD-L1 inhibitor	First line	MT	23	NA	NA	2.230(1.352-3.676)	0.002	7
					WT	113					
Daichi Fujimoto (Fujimoto et al., 2018)	2018	NSCLC	PD-1/PD-L1 inhibitor	First line	MT	95	1.45 (1.12-1.86)	0.006	NA	NA	7
					WT	410					
Jang Ho Cho (Cho et al., 2019)	2019	LUAD	Nivolumab	First line	MT	38	1.75(1.11-2.75)	0.02	NA	NA	7
					WT	140					

LUAD, lung adenocarcinoma; NSCLC, Non-small cell lung cancer; PFS, progression free survival; OS, overall survival; MT, mutant-type; WT, wild-type; HR, Hazard ratio; NA, Not available.

### Meta-Analysis Results

#### Comparison of KRAS, EGFR or TP53 Single Gene Mutation on PFS in Cancer Patients Using ICIs

**FIGURE 2:**
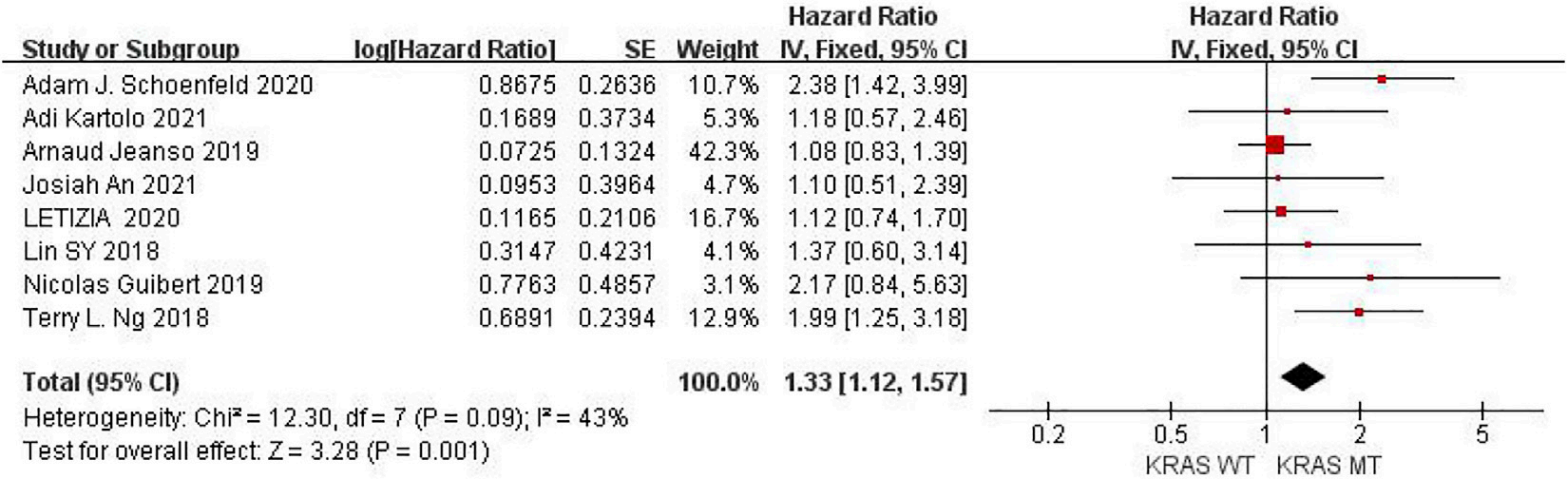
Meta-analysis of objective responses according to KRAS status of PFS in cancer patients treated with ICIs.

Eight studies can obtain KRAS single gene mutation. For the PFS data of cancer patients using ICIs, *I* ^*2*^ = 43%, *p* = 0.09. There is no statistical heterogeneity among the studies. The fixed effect model is used for analysis. The results showed that HR = 1.33 (95% CI = 1.12-1.57, *p* = 0.001), suggesting that KRAS single gene mutation can significantly improve PFS in cancer patients using ICIs, as shown in Figure 2.

**FIGURE 3:**
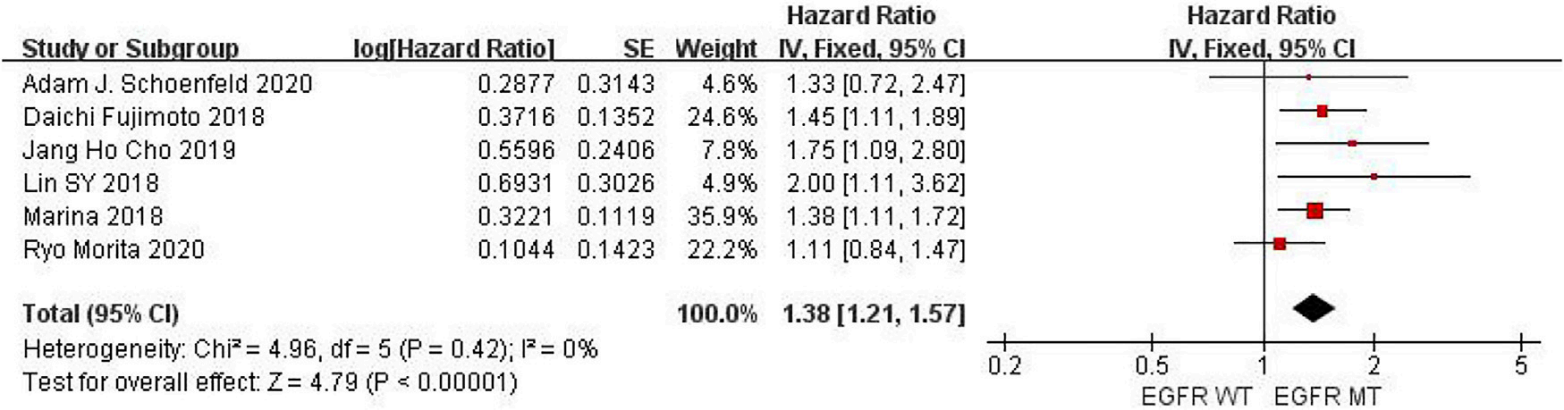
Meta-analysis of objective responses according to EGFR status of PFS in cancer patients treated with ICIs.

Six studies can obtain EGFR single gene mutation. For PFS data of cancer patients using ICIs, I^2^ = 0%, *p* = 0.42. There is no statistical heterogeneity among studies. Fixed effect model is used for analysis. The results showed that HR = 1.38 (95% CI = 1.21-1.57, *p* < 0.00001), suggesting that EGFR single gene mutation can significantly improve PFS in cancer patients using ICIs, as shown in Figure 3.

**FIGURE 4:**
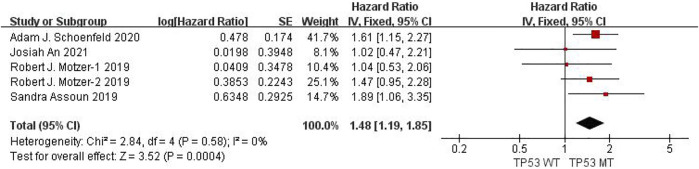
Meta-analysis of objective responses according to TP53 status of PFS in cancer patients treated with ICIs.

Six studies can obtain TP53 single gene mutation. The heterogeneity of PFS data of cancer patients using ICIs was analyzed, with *I* ^*2*^ = 0%, *p* = 0.58. There was no statistical heterogeneity among the studies. The fixed effect model was used for analysis. The results showed that HR = 1.48 (95% CI = 1.19-1.85, *p* = 0.0004), suggesting that TP53 single gene mutation can significantly improve PFS in cancer patients using ICIs, as shown in Figure 4.

The above univariate analysis results showed that KRAS, EGFR or TP53 single gene mutation can improve PFS, but the extended HRS is different. Compared with no mutation, the risk ratio of KRAS single gene mutation prolonging PFS is 1.33, EGFR single gene mutation prolonging PFS is 1.38, and TP53 single gene mutation prolonging PFS is 1.48. The results suggest that TP53 single gene mutation leads to more significant prolongation of PFS in cancer patients using ICIs.

### Comparison of KRAS, EGFR or TP53 on OS in Cancer Patients Using ICIs

**FIGURE 5:**
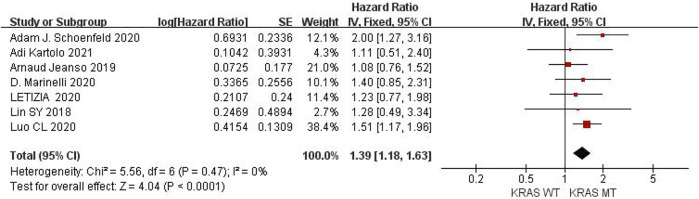
Meta-analysis of objective responses according to KRAS status of OS in cancer patients treated with ICIs.

Seven studies can obtain KRAS single gene mutation. For OS data of cancer patients using ICIs, I^2^ = 0%, *p* = 0.47. There is no statistical heterogeneity among studies. Fixed effect model is used for analysis. The results showed that HR = 1.39 (95% CI = 1.18-1.63, *p* < 0.00001), suggesting that KRAS single gene mutation can significantly improve the OS of cancer patients using ICIs, as shown in Figure 5.

**FIGURE 6:**
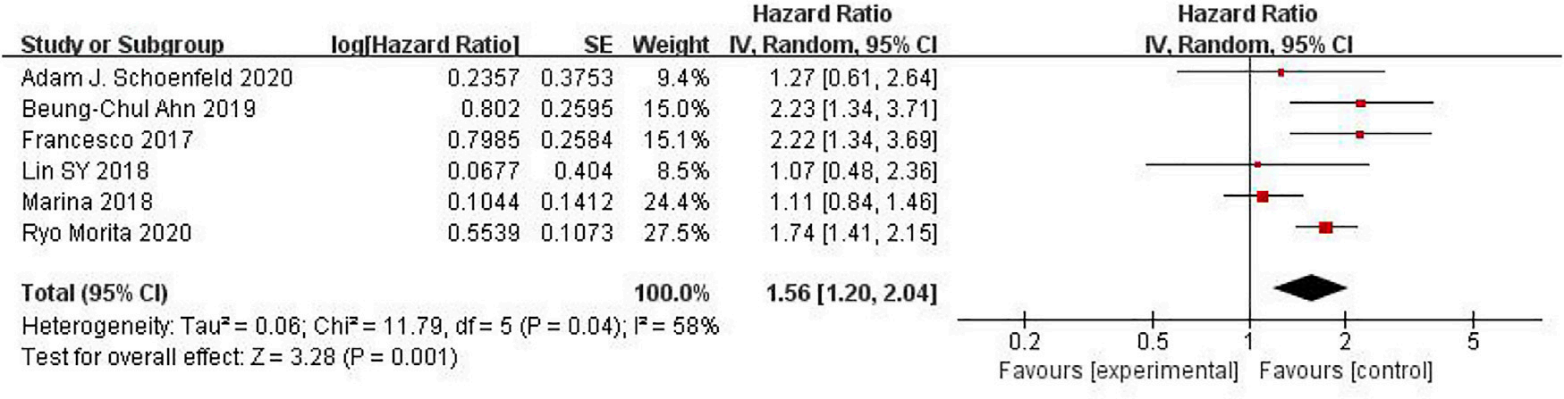
Meta-analysis of objective responses according to EGFR status of OS in cancer patients treated with ICIs.

Six studies can obtain EGFR single gene mutation. For OS data of cancer patients using ICIs, I^2^ = 58%, *p* = 0.04. There is statistical heterogeneity among studies, which is analyzed by random effect model. The results showed that HR = 1.56 (95% CI = 1.20-2.04, *p* = 0.001), suggesting that EGFR single gene mutation can significantly improve the OS of cancer patients using ICIs, as shown in Figure 6.

**FIGURE 7:**
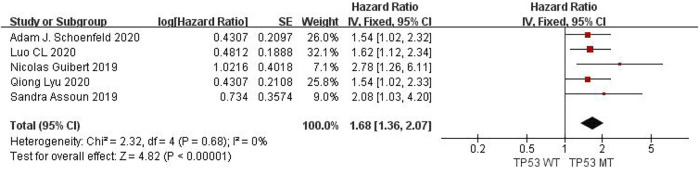
Meta-analysis of objective responses according to TP53 status of OS in cancer patients treated with ICIs.

Five studies can obtain TP53 single gene mutation. For OS data of cancer patients using ICIs, I^2^ = 0%, *p* = 0.68. There is no statistical heterogeneity among studies. Fixed effect model is used for analysis. The results showed that HR = 1.68 (95% CI = 1.36-2.07, *p* < 0.00001), suggesting that TP53 single gene mutation can significantly improve the OS of cancer patients using ICIs, as shown in Figure 7.

The above univariate analysis results show that KRAS, EGFR or TP53 single gene mutation can significantly improve OS, but the extended HRS is different. Compared with no mutation, the risk ratio of KRAS single gene mutation prolonging OS is 1.39, the risk ratio of EGFR single gene mutation prolonging OS is 1.56, and the risk ratio of TP53 single gene mutation prolonging OS is 1.68. The results suggest that TP53 single gene mutation leads to more significant OS prolongation in cancer patients using ICIs.

### Publication Bias

**FIGURE 8:**
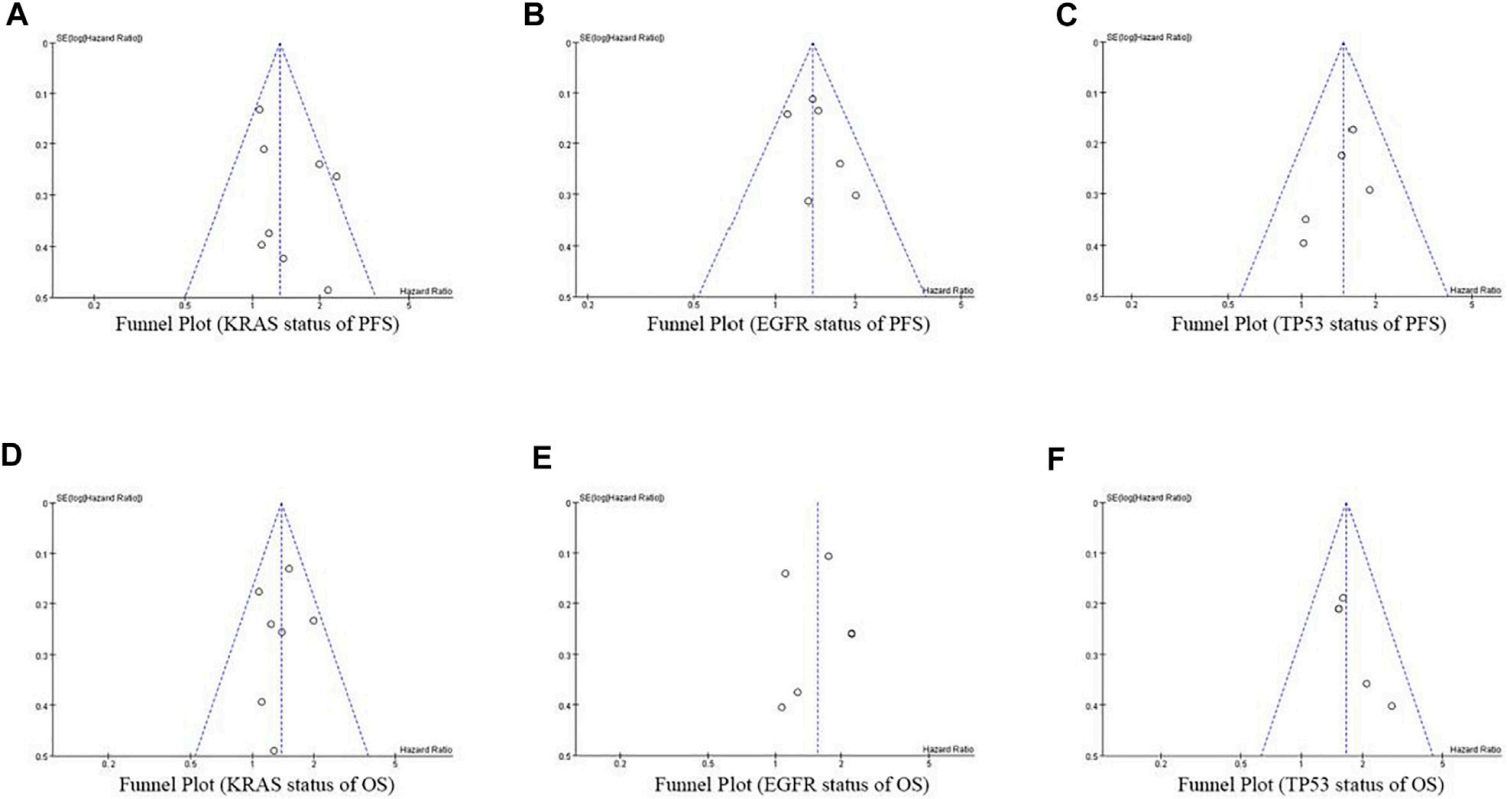
Funnel Plot of objective responses according to KRAS/EGFR/TP53 status of PFS and OS in cancer patients treated with ICIs.

At the same time of meta-analysis and comparison of PFS and OS data indicators, the inverted funnel diagram was drawn for the included studies. The results show that OS with EGFR single gene mutation has small publication bias. Individual studies may have less rigorous design and poor research methods, which lead to the asymmetry of inverted funnel diagram and small bias. Other PFS and OS funnel patterns are symmetrical, as shown in Figure 8.

### Sensitivity Analysis

In the sensitivity analysis, the sensitivity analysis of meta-analysis results was carried out by excluding one study at a time and then making statistics again. The combined hrs of PFS and OS showed no difference. There was no significant difference in the analysis results before and after elimination, suggesting that all meta-analysis results were stable.

## Discussion

A large number of research data show that the emergence of immune checkpoint inhibitors has significantly affected the clinical treatment strategies of most cancer subtypes, especially non-small cell lung cancer, renal cell carcinoma, melanoma and so on. Programmed cell death-1 (PD-1) inhibitors, such as neruzumab or pemilizumab, and programmed cell death ligand-1 (PD-L1) inhibitors, such as atezolizumab, significantly improved progression free survival (PFS) and overall survival (OS) in cancer patients. Compared with platinum based standard first-line chemotherapy or docetaxel based second-line chemotherapy, excellent disease control time is achieved (Borghaei et al., 2015; Le et al., 2017).

Some studies have shown that tumor mutation burden (TMB) can predict the potential activity of immunotherapy in a variety of tumor types, including NSCLC, RCC, etc. (Song et al., 2020). By producing damaged cell proteins recognized as new antigens by immune cells, the resulting genetic instability can enhance the immunogenicity of tumors, so as to optimize the antitumor cytotoxicity of T lymphocytes recovered by ICIs treatment. For example, in colorectal cancer, mismatch repair status predicts the clinical benefit of anti-PD-1 antibody pamumab (Van Allen et al., 2015). In non-small cell lung cancer, a mutation feature associated with smoking predicts anti-PD-1 efficacy (Hollern et al., 2019). In melanoma, TMB and neoantigen load predict patients’ response to CTLA-4 treatment (Pauken et al., 2016). The presence of CD8 + T cells and the expression of immune checkpoint genes such as PD-1 ligand (PD-L1) and CTLA-4 can also predict the efficacy of ICIs (Wang et al., 2019). These data show that TMB has a significant effect on predicting the response of ICIs, and it is clinically needed.

KRAS is a guanine nucleotide binding protein that regulates the mitogen activated protein kinase (MAPK) pathway. When it is activated, it promotes downstream signaling pathways, leading to cell growth and proliferation (Nagasaka et al., 2020). KRAS mutation rate in patients with non squamous NSCLC is 20–30% (Nagasaka et al., 2020). So far, there is no therapy for KRAS mutation. Although some targeted drugs (such as G12C inhibitors) are being evaluated in clinical trials, the effective drug targeting of KRAS mutation is also an unprecedented challenge. By studying the correlation between KRAS mutation status and TMB, it was found that TMB was associated with tumor immunogenicity and greater survival benefit of ICIs treatment (Adderley et al., 2019). Recent studies reported that compared with chemotherapy patients, OS and PFS were improved in patients with KRAS mutant NSCLC after ICIs treatment, and KRAS mutant tumors showed stronger PD-L1 expression and T cell infiltration (Adderley et al., 2019). Epidermal growth factor receptor (EGFR) mutation is a good predictor of the efficacy of EGFR tyrosine kinase inhibitors. EGFR mutation is common in cancer, especially in non-small cell lung cancer. EGFR mutations are found in 32% of non-small cell lung cancer cases worldwide, of which 39% of tumors occur in Asian ethnic groups and 17% in Caucasian ethnic groups (Zhang et al., 2016). The prevalence of EGFR mutation in lung adenocarcinoma is high; 51% of East Asian lung adenocarcinoma patients have EGFR mutation (Shi et al., 2014). The high prevalence of EGFR mutations makes it more important to evaluate whether EGFR mutations are really negative predictors of PD-1/PD-L1 efficacy. The prognostic role of tumor suppressor gene TP53 mutation in predicting the efficacy of ICIs is highly controversial. An early study (Xiao et al., 2018) showed that TP53 mutation was associated with relatively short PFS and shorter OS in 110 patients receiving CTLA-4 blocking treatment. However, inconsistent results were observed in another group of patients with non-small cell lung cancer treated with PD-1 + CTLA-4 block. The study found that TP53 mutation was enriched in responders, suggesting that TP53 may be related to the enhanced response of combination therapy. In addition, Dong et al. (Ozaki et al., 2020) confirmed that patients with lung adenocarcinoma had a relatively good response to TP53 mutation when treated with PD-1 inhibitors, suggesting that TP53 may help to guide the decision of clinical use of ICIs.

In addition, many cancers show different patterns of genomic changes. In the development of cancer, KRAS, as a driving oncogene, has a high mutation frequency. However, it has recently been found that cancers with KRAS mutations also express TP53 mutations. TP53, as a core cancer suppressor gene, encodes p53 protein in humans and mice to prevent mutations in a stable state. Notably, TP53 mutations drive lung cancer, with a higher frequency of TP53 mutations than EGFR or KRAS mutations in LUAD. Studies have shown that tumors with co-mutations of KRAS/TP53 usually show significant upregulation of PD-L1 expression and accumulation of tumor-killing T cells. In this regard, in addition to PD-L1 expression, TP53 mutations will hopefully guide the clinical application of immune checkpoint blocking therapy for KRAS mutated lung adenocarcinoma. However, the prognostic effect of TP53 mutation on EGFR mutant lung cancer is controversial. TP53/EGFR co-mutations may be associated with treatment resistance and shorter survival in lung cancer patients, the study showed. On the other hand, Labbe et al. (Zhang et al., 2019) studied 105 egFR-mutant NSCLC patients, and among the patients undergoing surgery, progressionfree survival (HR 0.99, 95%CI: 0.56-1.75) and overall survival (OS, HR 1.39, 95%CI: 0.70-2.77) was unrelated to TP53 status. Therefore, considering these controversial findings and evaluating the prognostic value of TP53/EGFR co-mutations in lung cancer will also be the focus of our subsequent studies.

In this meta-analysis, we investigated whether the survival benefits of ICIs in cancer patients vary according to KRAS/EGFR/TP53 single gene mutation status. We found that the PFS and OS of patients with KRAS/EGFR/TP5 single gene mutant tumors were significantly improved and the degree of improvement was different when treated with ICIs. TP53 mutant showed a better effect than KRAS mutation and EGFR mutation. These results suggest that KRAS/EGFR/TP53 single gene mutation status may be a potential biomarker of the survival benefits of ICIs. In the future, ICIs may play an important role in the treatment of early cancer. After radiotherapy and chemotherapy, immunotherapy has become the standard treatment of cancer, regardless of its mutation status. Even in the early stages of the disease, molecular characteristics will become the basis to determine the optimal treatment strategy and its combination with local regional treatment, especially in oncogene addiction diseases.

Considering the limited amount of data based on this meta-analysis, further research is needed to evaluate the effect of KRAS/EGFR/TP53 single gene mutation on the efficacy of ICIs in cancer patients. We also expect that the latest progress of next-generation sequencing technology will help to identify more accurate biomarkers and bring clinical benefits to ICIs immunotherapy.

## Data Availability

The original contributions presented in the study are included in the article/Supplementary Material, further inquiries can be directed to the corresponding author.
